# Design of Metamaterial Sensor for Non Destructive Testing of Aircraft Composite Skin Damage

**DOI:** 10.3390/mi16030284

**Published:** 2025-02-28

**Authors:** Zhaoxuan Zhu, Rongqing Kang, Kaiyu Qin

**Affiliations:** School of Aeronautics and Astronautics, University of Electronic Science and Technology of China, Chengdu 611731, China; 202122100138@std.uestc.edu.cn (R.K.); kyqin@uestc.edu.cn (K.Q.)

**Keywords:** nondestructive detection, aircraft composite skin, metamaterial sensor

## Abstract

The detection of aircraft skin is an important part of the process of aircraft design, manufacturing, and application. This paper proposes a metamaterial sensor for non-destructive detection of aircraft composite skin damage. Firstly, Using the perturbation theory, an electromagnetic nondestructive detection model of aircraft composite skin is established. Then, according to the plasmon theory, a nested multi-layer complementary split resonant ring (CSRR) metamaterial sensor is designed. Simulation using the high frequency structure simulator (HFSS), it shows that it can effectively detect defect with diameters of 2 mm and above and burial depth within 3 mm. Finally, the physical test is carried out, and the 10 mm diameter defect in the aircraft composite skin sample can be detected.

## 1. Introduction

Carbon fiber reinforced polymer (CFRP) has the advantages of light weight [1], high compressive strength [2], and fatigue resistance [3], and is widely used in the field of aircraft [4]. However, composite materials are damaged during use due to impact, such as surface dents, delamination, matrix cracking, fiber breakage, etc., which will directly affect the strength, stiffness, fatigue life and other properties of the material, causing safety hazards. Therefore, the detection of defects in aircraft composite skin is extremely important.

Nowadays, the non-destructive testing (NDT) methods for aircraft composite skin mainly include the following: infrared thermal imaging [5], ultrasonic testing [6], X-ray testing [7] and eddy current testing [8]. However, ultrasonic testing requires coupling agents, eddy current testing incapable of detecting metallic materials and has low resolution, infrared thermal imaging can easily damage the surface of an aircraft, X-ray testing lack convenience. In order to overcomes the shortcomings of traditional testing method, The metamaterial sensor is proposed for Nondestructive testing of aircraft composite skin.

Split resonant ring (SRR) and its complementary structure are often used in near-field microwave non-destructive testing due to their good electromagnetic field local characteristics [9,10]. Albishi, A. M used a complementary split ring resonator fed by a microstrip transmission line as a sensor to detect cracks on a metal surface covered or hidden by a dielectric layer [11]. Zhen Li proposed a method for impact damage detection of carbon fiber based on a complementary split ring resonator sensor [12]. The traditional CSRR structure has problems such as high resonant frequency and unfocused energy. Therefore, this paper improves on the CSRR structure and proposes a nested multi-layer CSRR structure, which maintains a more focused energy and has a lower operating frequency than the CSRR structure of the same size. 

This paper is organized as follows. We investigate the model of aircraft composite skin defects in Section 2. In Section 3, nested multi-layer CSRR metamaterial sensor is designed. The simulation and experiment are shown in Section 4 and finally, we present our conclusions about the work in Section 6.

## 2. Model of Aircraft Composite Skin Defect

**Figure 1:**
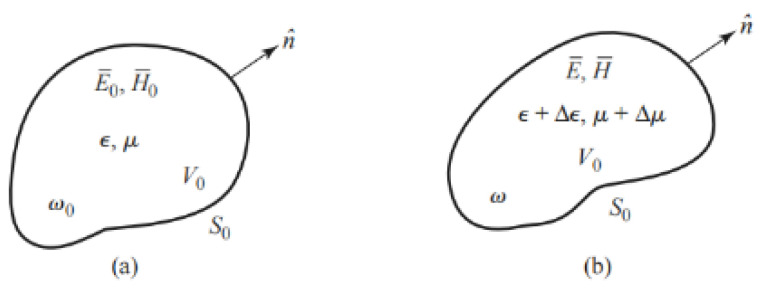
Change in dielectric constant or permeability (**a**) the original resonant cavity, (**b**) the disturbed resonant cavity.

The detection of aircraft composite skin using CSRR can be equivalent to a resonator. The resonator loaded with the sample to be tested is considered as a complete resonator, and the resonant frequency is disturbed by the defect of the sample to be tested, which can be analyzed based on perturbation theory [13]. A resonator that is disturbed by a change in dielectric constant or permeability is shown in Figure 1.

If ${\bar{E}}_{0}$, ${\bar{H}}_{0}$ is the field of the original resonant cavity, while $\bar{E}$, $\bar{H}$ The field in the disturbed resonant cavity. So, the Maxwell rotation equations in these two cases are shown in Equations (1), (2), (3), and (4), respectively.

(1) $$\nabla \times {\bar{E}}_{0}=-j\omega_{0}\mu {\bar{H}}_{0}$$

(2) $$\nabla \times {\bar{H}}_{0}=j\omega_{0}\epsilon {\bar{E}}_{0}$$

(3) $$\nabla \times \bar{E}=-j\omega \left(\mu +\Delta \mu \right)\bar{H}$$

(4) $$\nabla \times \bar{H}=j\omega \left(\epsilon +\Delta \epsilon \right)\bar{E}$$

where, $\omega_{0}$ is the resonant frequency of the original resonant cavity, ω  is the resonant frequency of the perturbation cavity, Δϵ and Δμ are the change in material properties inside the resonant cavity caused by defects.

Multiplying the conjugate of Equation (1) by $\bar{H}$ and Equation (4) by the conjugate of ${\bar{E}}_{0}$ yields Equations (5) and (6), respectively.

(5) $$\bar{H}\cdot \nabla \times {\bar{E}}_{0}^{*}=j\omega_{0}\mu \bar{H}\cdot {\bar{H}}_{0}^{*}$$

(6) $${\bar{E}}_{0}^{*}\cdot \nabla \times \bar{H}=j\omega \left(\epsilon +\Delta \epsilon \right){\bar{E}}_{0}^{*}\cdot \bar{E}$$

where, ${\bar{E}}_{0}^{*}$ is the conjugate of ${\bar{E}}_{0}$, ${\bar{H}}_{0}^{*}$ is the conjugate of ${\bar{H}}_{0}$.

Subtracting Equation (6) from Equation (5), combined with vector transformation formula $\nabla \cdot \left(\bar{A}\times \bar{B}\right)=\bar{B}\cdot \nabla \times \bar{A}-\bar{A}\cdot \nabla \times \bar{B}$, yields Equation (7).

(7) $$\nabla \cdot \left({\bar{E}}_{0}^{*}\times \bar{H}\right)=j\omega_{0}\mu \bar{H}\cdot {\bar{H}}_{0}^{*}-j\omega \left(\epsilon +\Delta \epsilon \right){\bar{E}}_{0}^{*}\cdot \bar{E}$$

Similarly, multiplying the conjugate of Equation (2) by $\bar{E}$ and Equation (3) by the conjugate of ${\bar{H}}_{0}$ yields Equations (8) and (9), respectively.

(8) $$\bar{E}\cdot \nabla \times {\bar{H}}_{0}^{*}=-j\omega_{0}\epsilon {\bar{E}}_{0}^{*}\cdot \bar{E}$$

(9) $${\bar{H}}_{0}^{*}\cdot \nabla \times \bar{E}=-j\omega \left(\mu +\Delta \mu \right){\bar{H}}_{0}^{*}\cdot \bar{H}$$

Subtracting Equation (8) from Equation (9), combined with vector transformation formula $\nabla \cdot \left(\bar{A}\times \bar{B}\right)=\bar{B}\cdot \nabla \times \bar{A}-\bar{A}\cdot \nabla \times \bar{B}$, yields Equation (10).

(10) $$\nabla \cdot \left(\bar{E}\times {\bar{H}}_{0}^{*}\right)=-j\omega \left(\mu +\Delta \mu \right){\bar{H}}_{0}^{*}\cdot \bar{H}+j\omega_{0}\epsilon {\bar{E}}_{0}^{*}\cdot \bar{E}$$

Add Equation (7) and Equation (10), then integrate them over the volume $V_{0}$, and combine the divergence theorem to obtain Equation (11).

(11) $$\begin{array}{ll} & \;\int_{V_{0}}\nabla \cdot \left({\bar{E}}_{0}^{*}\times \bar{H}+\bar{E}\times {\bar{H}}_{0}^{*}\right)dv\\ & =j\int_{V_{0}}\left\{\left[\omega_{0}\epsilon -\omega (\epsilon +\Delta \epsilon )\right]{\bar{E}}_{0}^{*}\cdot \bar{E}+\left[\omega_{0}\mu -\omega (\mu +\Delta \mu )\right]{\bar{H}}_{0}^{*}\cdot \bar{H}\right\}dv=0 \end{array}$$

where, $V_{0}$ is the volume of the resonant cavity.

By organizing Equation (11), Equation (12) can be obtained.

(12) $$\frac{\omega -\omega_{0}}{\omega }=\frac{-\int_{V_{0}}\left(\Delta \epsilon \bar{E}\cdot {\bar{E}}_{0}^{*}+\Delta \mu \bar{H}\cdot {\bar{H}}_{0}^{*}\right)dv}{\int_{V_{0}}\left(\epsilon \bar{E}\cdot {\bar{E}}_{0}^{*}+\mu \bar{H}\cdot {\bar{H}}_{0}^{*}\right)dv}$$

If Δϵ and Δμ are small, the original field ${\bar{E}}_{0}$ and ${\bar{H}}_{0}$ can be approximated as the perturbation field $\bar{E}$ and $\bar{H}$, and $\omega_{0}$ can be approximated as *ω*. Equation (12) is transformed into Equation (13).

(13) $$\frac{\omega -\omega_{0}}{\omega_{0}}≃\frac{-\int_{V_{0}}\left(\Delta \epsilon {\left|{\bar{E}}_{0}\right|}^{2}+\Delta \mu {\left|{\bar{H}}_{0}\right|}^{2}\right)dv}{\int_{V_{0}}\left(\epsilon {\left|{\bar{E}}_{0}\right|}^{2}+\mu {\left|{\bar{H}}_{0}\right|}^{2}\right)dv}$$

Based on the above analysis, changes in the dielectric constant and magnetic permeability in a resonant cavity will cause changes in the resonant frequency. Therefore, by detecting the change in resonant frequency, the changes in dielectric constant and magnetic permeability can be detected.

The metamaterial sensor designed in this paper is attached to the surface of an aircraft skin. The coverage area of the metamaterial sensor is equivalent to a resonant cavity. Damage to the aircraft skin causes a change in the dielectric constant in the resonant cavity, resulting in a shift in the resonant frequency of the metamaterial sensor. Implement the detection of aircraft skin damage.

## 3. Design of Nested Multi-Layer CSRR Metamaterial Sensor

**Figure 2:**
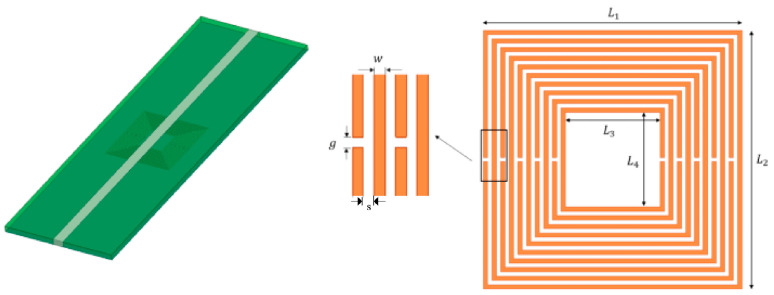
Nested multi-layer CSRR metamaterial sensor.

According to reference [14], the dielectric constant of aircraft composite skin was obtained through experimental testing, and its resonant frequency was approximately 0.9 GHz. Therefore, the resonant frequency of metamaterial sensor is selected as 0.9 GHz. The proposed nested multi-layer CSRR metamaterial sensor structure is shown in Figure 2. The microstrip transmission line substrate is FR4-epoxy with a thickness of 0.1 mm, its relative dielectric constant is 4.4, the loss tangent is 0.02, and the size is 60 mm × 20 mm. The metal conductor strip width of the microstrip transmission line is 1.86 mm to achieve 50-ohm impedance matching. The metal strip and ground plate material of the microstrip transmission line are both copper, with a thickness of 35 um. Next, design and analyze the shape of the metasurface sensor, the width of the ring gap, the width of the metal ring, the edge length of the outermost ring, and the number of nested layers to obtain the optimal aircraft composite skin detection metasurface sensor.

**Figure 3:**
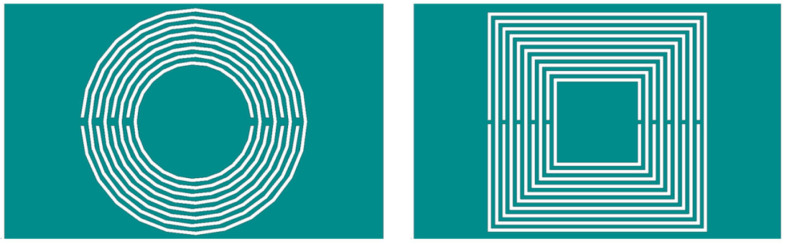
Square and circular nested multi-layer CSRR (l = 12 mm, w = 0.2 mm, g = s = 0.2 mm, N = 10).

**Figure 4:**
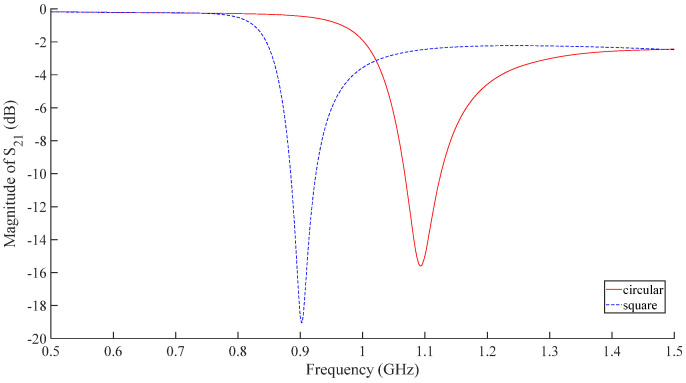
S_21_-curve of sensors with square and circular shapes.

Design circular and square metasurface sensors with identical ring gap width, metal ring width, outermost ring edge length or diameter, and nesting layers. Use HFSS to design two types of shape sensors as shown in Figure 3 and simulate them to obtain the frequency response of their S_21_ parameters as shown in Figure 4. According to the analysis of Figure 4, the resonant frequency of the circular structure is 1.093 GHz, while the resonant frequency of the square structure is 0.9025 GHz. Therefore, the resonant frequency of the square structure with the same size is more suitable for aircraft composite skin detection compared to the circular structure.

**Figure 5:**
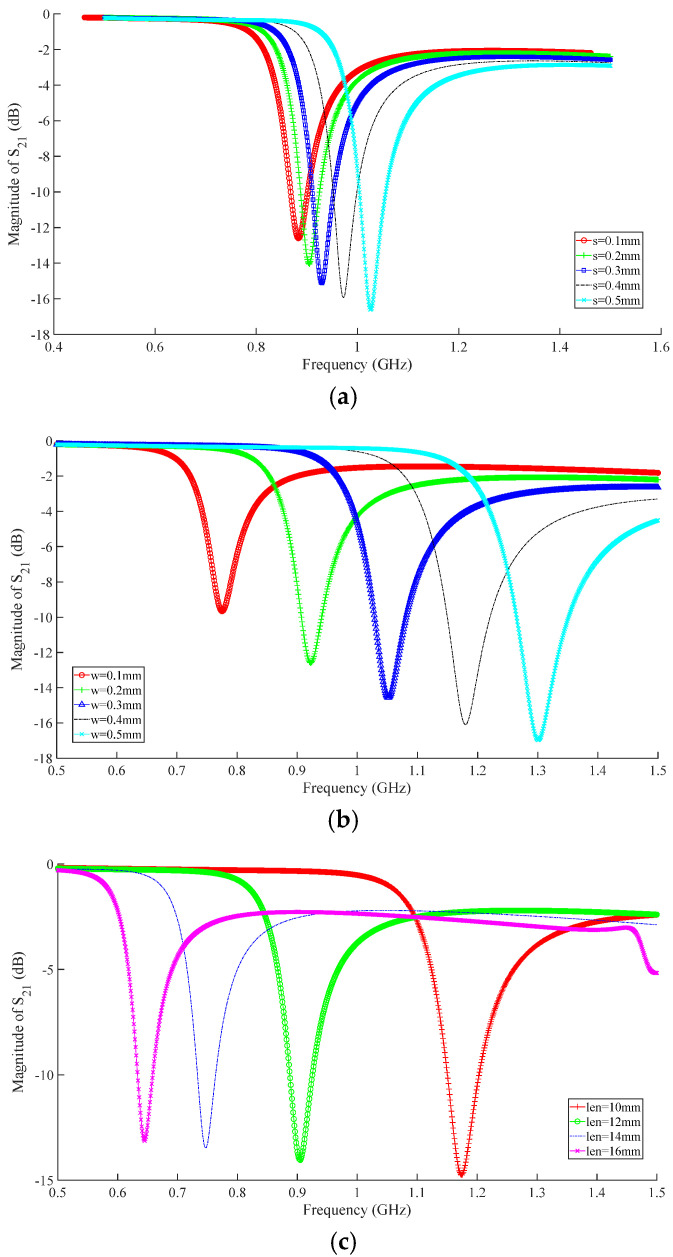
S_21_-curves of different structural sizes (**a**) different gap widths, (**b**) different metal ring widths, (**c**) different outer ring edge lengths.

Using a square structure, the S_21_ frequency response was analyzed using HFSS for different parameters such as metal ring width, outermost ring edge length, and nesting layers. The simulation results are shown in Figure 5.

According to the analysis of Figure 5a, when g = s = 0.2 mm, the resonant frequency is 0.894 GHz. At the same time, when the ring gap width is between 0.3 mm and 0.5 mm, the resonant frequency of the resonant structure is more sensitive to changes in the ring gap width, with a sensitivity of up to 0.53 GHz/mm. When the ring gap width is less than 0.3 mm, the sensitivity of the resonant frequency to the ring gap width is 0.25 GHz/mm.

The width of the metal ring w varies from 0.1 mm to 0.5 mm with a step size of 0.1 mm. The S_21_ curve is shown in Figure 5b. As w increases, the resonant frequency of the sensor increases. When w = 0.2 mm, the resonant frequency is 0.9075 GHz.

The side length of the resonant structure changes from 10 mm to 16 mm, with a step size of 2 mm. The S_21_ curve is shown in Figure 5c. As it increases, the resonant frequency of the sensor decreases. When l = 10 mm, the resonant frequency is 1.174 GHz, while when l = 16 mm, the resonant frequency shifts to 0.645 GHz. When l = 12 mm, the resonant frequency is 0.9002 GHz.

Therefore, when s = 0.2 mm, w = 0.2 mm, and l  = 12 mm, the resonant frequency is more suitable for aircraft composite skin detecting.

**Figure 6:**
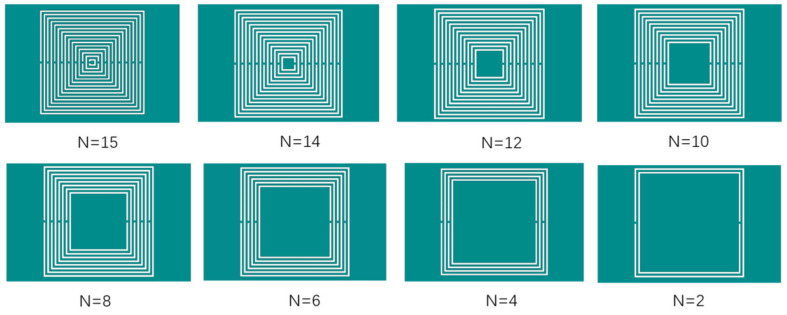
Nested multi-layer CSRR structure with different nesting layers (l = 12 mm, w = 0.2 mm, g = s = 0.2 mm).

**Figure 7:**
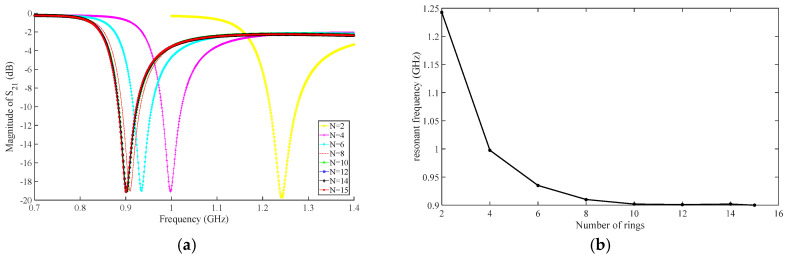
(**a**) Frequency response curves of S_21_-parameters with different nesting levels; (**b**) Relationship between resonant frequency and nested layers.

The number of nested layers varies from 2 to 15, while parameters such as the outermost ring radius, metal ring width, and gap width remain unchanged. The structure is shown in Figure 6. Simulation and analysis are conducted using HFSS software, and the simulation results are shown in Figure 7. As the number of nested layers increases, the resonant frequency begins to significantly decrease and then tends to remain unchanged. When N = 2, the resonant frequency is 1.2425 GHz, decreasing at a rate of 0.25 GHz/mm. When N = 10, the resonant frequency is 0.9025 GHz, and as the number of embedded rings increases, the resonant frequency remains basically unchanged.

Based on the above analysis, when choosing a square sensor with s = 0.2 mm, w = 0.2 mm, l = 12 mm, and N = 10, the metasurface sensor operates at approximately 9 GHz. More suitable for aircraft composite skin testing. Therefore, the dimensions of the metasurface sensor are shown in Table 1.

**Figure 8:**
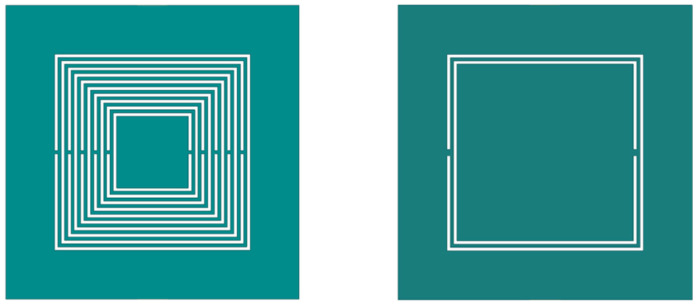
Nested multi-layer CSRR Structure and Traditional CSRR Structure.

**Figure 9:**
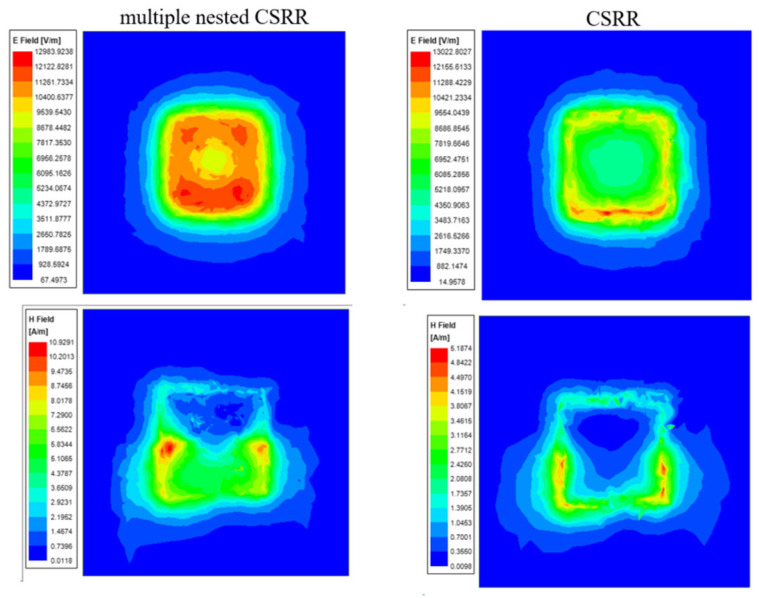
Electric and magnetic field distributions of nested multi-layer CSRR structure and traditional CSRR structure.

**Figure 10:**
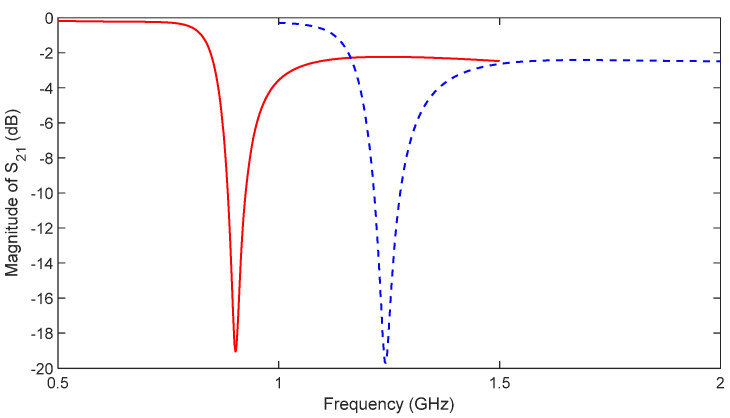
S_21_-curve of nested multi-layer CSRR structure and traditional CSRR structure (“red line” represents multi-level CSRR, “blue line” represents traditional CSRR).

Compared with traditional CSRR metasurface sensors, nested multi-layer CSRR metasurface sensor with s = 0.2 mm, w = 0.2 mm, l = 12 mm, and N = 10 is designed, as well as traditional CSRR metasurface sensor with s = 0.2 mm, w = 0.2 mm, and l = 12 mm. Two types of sensing structures are shown in Figure 8, and the HFSS software is used to simulate the two structures. The distribution of electric and magnetic field strength at 1 mm directly above the resonant structure is shown in Figure 9. Analysis of Figure 8 shows that the nested CSRR structure increases the number of layers, resulting in more concentrated electric and magnetic field strength. The traditional CSRR structure is more dispersed in the sensing area. At the same time, the frequency response of two structures S_21_ is analyzed, and the simulation results are shown in Figure 10. The resonant frequency of the traditional CSRR metasurface sensor is 1.242 GHz, while the resonant frequency of the nested multi-layer CSRR metasurface sensor is 0.902 GHz. The nested multi-layer CSRR metasurface sensor is more suitable for aircraft composite skin detecting.

**Figure 11:**
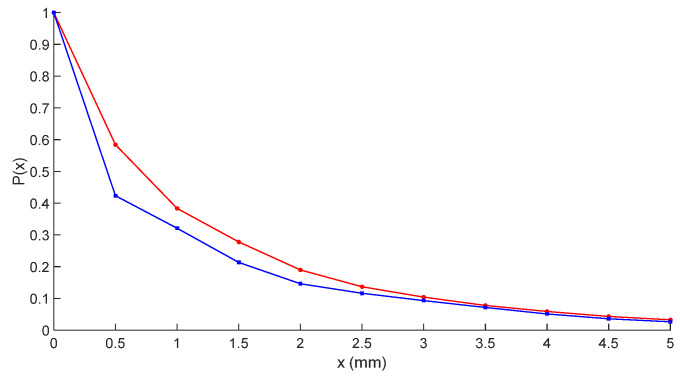
Relationship between energy attenuation and distance between nested multi-layer CSRR structure and traditional CSRR structure (“red line” represents multi-level CSRR, “blue line” represents traditional CSRR).

Similarly, using the aircraft composite skin test object described in Section 4, analyze the attenuation depth of two sensors. The energy attenuation is shown in Figure 11, where x represents depth in millimeters and P (x) is the normalized energy value. The nested multi-layer CSRR structure sensor has lower energy attenuation in the range of 0 mm to 3 mm compared to traditional CSRR structures. Within the depth range of 0 mm to 3 mm, the nested multi-layer CSRR structure sensor has better defect detection performance than traditional CSRR structures.

## 4. Simulation and Analysis of Aircraft Composite Skin Detection

### 4.1. Simulation Model

**Figure 12:**
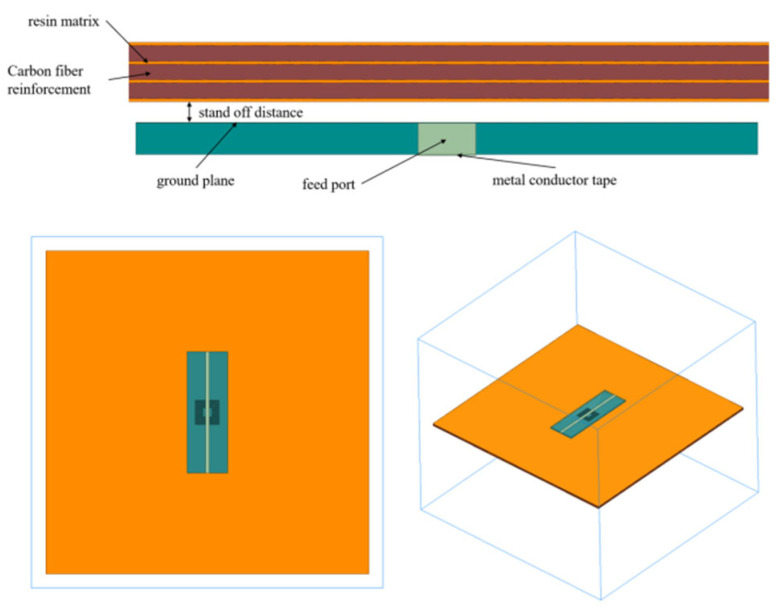
Aircraft composite skin defect detection simulation model.

In engineering applications, the aircraft composite skin is usually made by stacking multiple layers of pre-impregnated fabric in specific orientations and layer counts. Therefore, a single layer of pre-impregnated fabric often serves as the fundamental unit of the three-dimensional model [15]. The aircraft composite skin sample model was constructed, as shown in Figure 12, which is a three-layer orthogonal plate with a thickness of 1.9 mm, comprising three layers of carbon fiber reinforcements and four layers of resin matrix. The thin plates of resin matrix are used to simulate the resin accumulation between the layers of layup. The thickness of the carbon fiber reinforcement thin plates is set at 0.5 mm, and the thickness of the resin matrix thin plates is set at 0.1 mm. Assuming the fiber direction parallel to the y-axis is 0°, the fiber orientations of the three layers of carbon fiber reinforcements are [0°, 90°, 0°]. The relative permittivity and conductivity of the carbon fiber reinforced plate are set as anisotropic tensors [16], as shown in Table 2. The microstrip sensor is positioned directly above the aircraft composite skin, maintaining 1 mm. The model uses a finite air domain to simulate an unbounded free space, with the air domain boundary conditions set as absorbing boundaries.

### 4.2. Detection of Internal Defects in Aircraft Composite Skin

**Figure 13:**
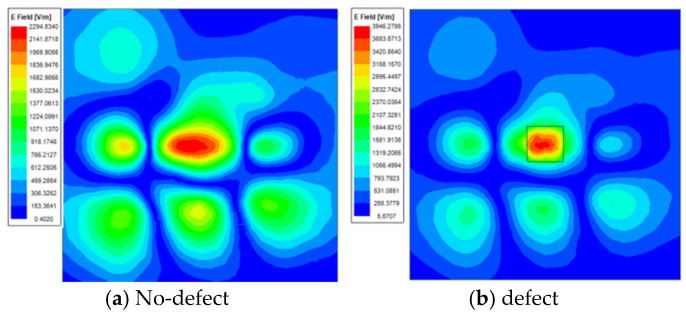
Electric field distribution diagram.

The defect in the aircraft composite skin is a 5 mm × 5 mm × 0.05 mm, located within the resin matrix between the second and third layers of fiber boards, directly beneath the resonant structure of the sensor. Figure 13a displays the distribution of the electric field inside the aircraft composite skin sample when there are no defects. Figure 13b shows the electric field distribution inside the aircraft composite skin sample when it has damage. It is evident from the Figure 13 that there is a significant change in the electric field distribution at the defect site, not only is the intensity of the electric field at the defect site significantly stronger compared to areas without defects, but it is also more concentrated around the area of the defect. The change in the reflection coefficient is related to the field disturbance at the target defect site before and after the defect.

**Figure 14:**
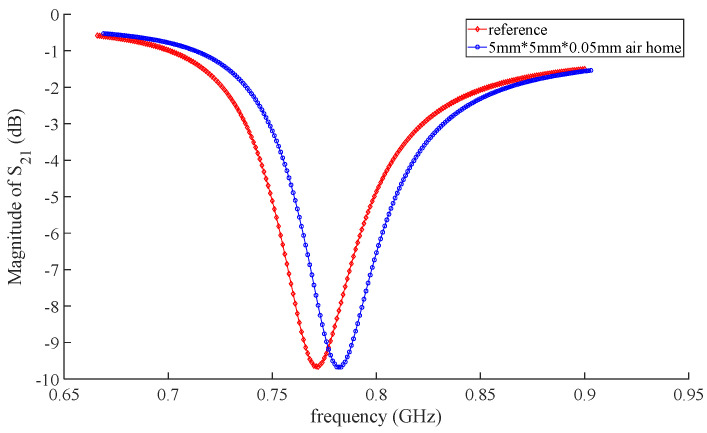
The frequency shift with defects and no defect.

Testing aircraft composite skin samples with and without defects, the frequency response of sensor S_21_ parameters is shown in Figure 14. From Figure 14, it can be observed that compared to the no-defect aircraft composite skin sample, the aircraft composite skin sample with internal defects shifts the sensor’s resonant frequency from 0.772 GHz to 0.782 GHz. Compared to the resonant frequency, the amplitude of the S_21_ coefficient remains essentially unchanged. The frequency shift can effectively identify internal damage in aircraft composite skin.

**Figure 15:**
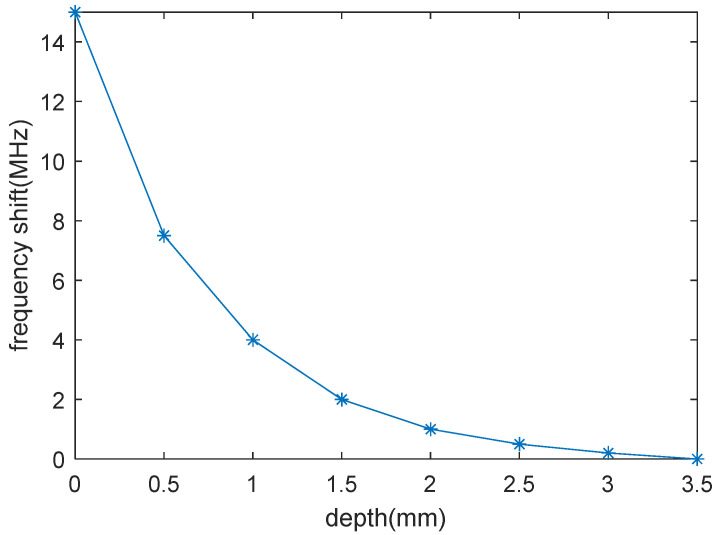
The frequency shift with defect depth.

At the same time, the frequency shift of the simulation test defect at different depths of the aircraft skin is shown in Figure 15. Analysis of Figure 15 shows that the frequency shift decreases with increasing defect depth, and when the depth exceeds 3 mm, the frequency shift is difficult to distinguish.

**Figure 16:**
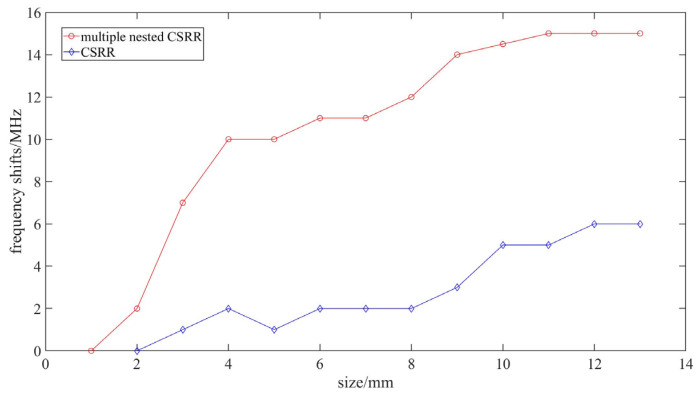
Relationship between resonant frequency shift and defect size between nested multi-layer CSRR metasurface sensor and traditional CSRR metasurface sensor.

In order to evaluate the detection resolution of nested multi-layer CSRR metasurface sensor, simulations are conducted in HFSS for defects of different sizes. The frequency shift of the nested multi-layer CSRR metasurface sensor is shown as the “red line” in Figure 16. Analysis of Figure 16 shows that when the diameter of the defect is 1 mm or smaller, the resonant frequency shift of the nested multi-layer CSRR sensor is about zero. When the size is greater than or equal to 2 mm, there is a significant resonant frequency shift, and as the defect size increases, the resonant frequency shift of the sensor increases. Compare with the traditional CSRR structure sensor. The resolution of traditional CSRR structure sensors is shown by the “blue line”in Figure 16, and the resolution of traditional CSRR structure sensors is above 4 mm.

**Figure 17:**
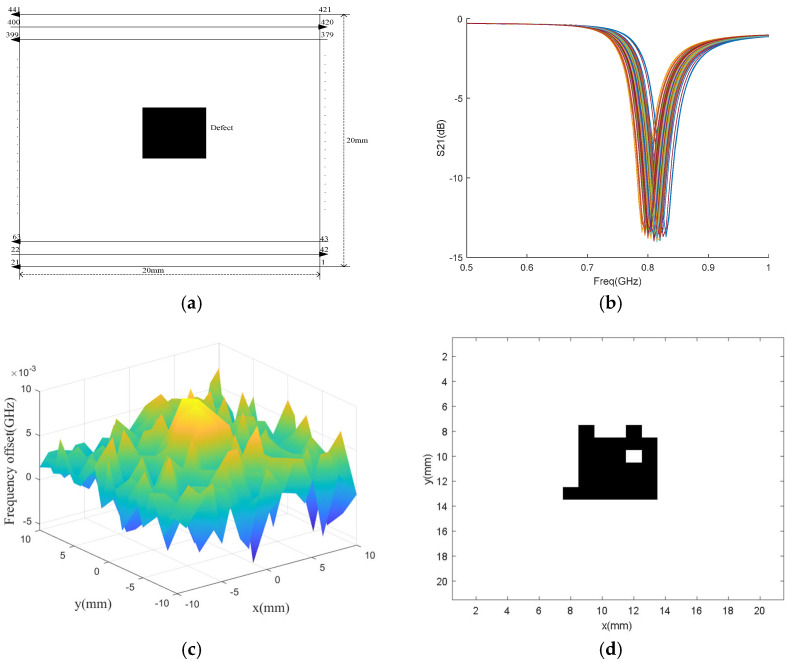
Defect detection of aircraft composite skin samples. (**a**) Scanning process and defect distribution; (**b**) Scan the S_21_ parameter frequency response at each point of the sample; (**c**) Scan the three-dimensional frequency offset map of each point; (**d**) Processed frequency offset black and white image.

Using nested multi-layer CSRR metasurface sensor, the aircraft composite skin sample with dimensions of 20 mm × 20 mm, thickness of 1.9 mm, defect located at the center, size of 5 mm × 5 mm × 0.05 mm, and depth of 0.6 mm is scanned. The scanning step is 1 mm, and a total of 441 points are scanned. The scanning and defect distribution are shown in Figure 17a. The S_21_ frequency response of each scanning point is obtained using the HFSS simulation software, as shown in Figure 17b. The frequency offset of each scanning point is calculated, as shown in Figure 17c. Using principal component analysis to process frequency offset, that is, firstly covariance the scanned frequency data to obtain the covariance matrix; Then calculate the eigenvalues and eigenvectors of the covariance matrix to obtain the covariance matrix of the principal components; Finally, based on principal component reconstruction data, defect recognition is performed. The defects of the aircraft composite skin sample obtained is shown in Figure 17d. Analysis of Figure 17d shows that nested multi-layer CSRR metasurface sensor can effectively detect defect in the aircraft composite skin sample.

Compared with other detection methods, the comparison results are shown in Table 3. The nested CSRR structure metamaterial sensor exhibits certain advantages, with better resolution than other detection methods.

## 5. Experiment

### 5.1. Experimental Platform

**Figure 18:**
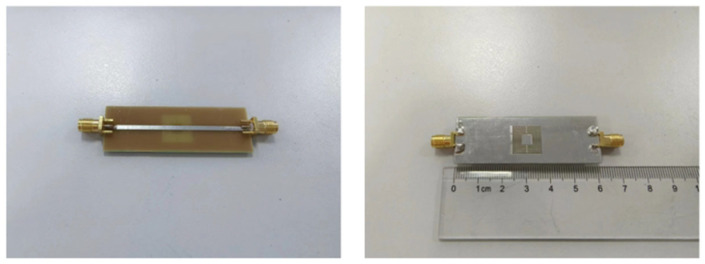
Nested Multi Layer CSRR Metasurface Sensor in Physical Form.

**Figure 19:**
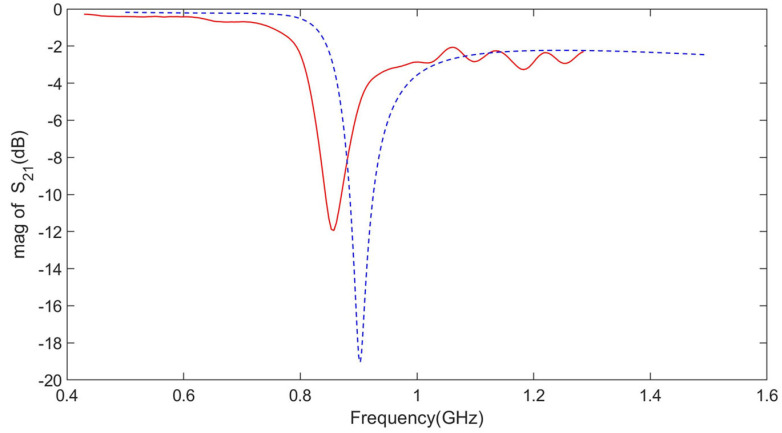
Nested multi-layer CSRR metasurface sensor S_21_ parameter measurement and simulation frequency response(“red line” represents test “blue line” represents simulation).

Design and manufacture nested multi-layer CSRR metasurface sensor according to the dimensions in Section 3, as shown in Figure 18. Using a vector network analyzer to test the number of sensor parameters, the S_21_ parameter frequency response was obtained as shown by the “red line” in Figure 19, and the simulation data is shown by the “blue line” in Figure 19. There is a certain gap between the resonant frequency and the simulation results, which is due to the introduction of some additional parasitic capacitance and inductance at the welding point. However, the resonator frequency is within the resonant frequency of the aircraft composite skin and does not affect the detection of defects in the aircraft composite skin.

**Figure 20:**
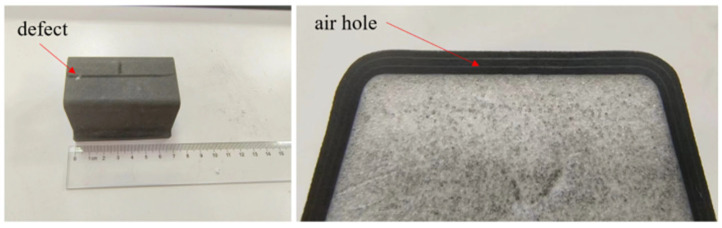
Physical aircraft composite skin sample.

The tested aircraft composite skin sample is shown in Figure 20, with dimensions of 30 mm × 67 mm × 1.5 mm. Create a defect with a diameter of 10 mm at the white mark shown in Figure 20, located at a depth of 0.6 mm. In practical engineering, defects larger than 10 mm are usually defined as faults. Therefore, the 10 mm defect is selected for testing and verification.

**Figure 21:**
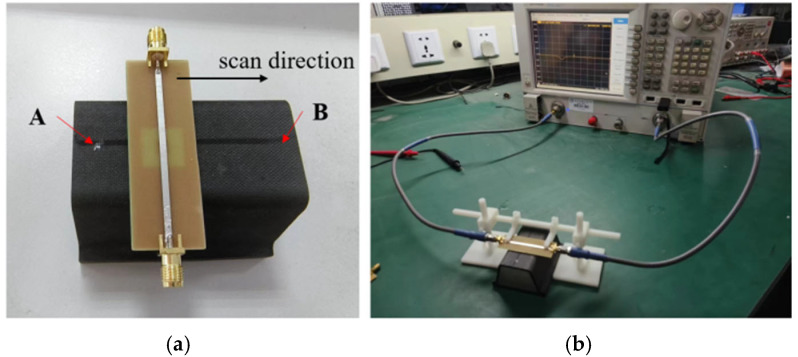
Scanning Test Platform. (**a**) Linear scanning process; (**b**) Test Platform.

As shown in Figure 21a, the nested multi-layer CSRR Metasurface sensor is placed directly above the aircraft composite skin sample, with the center of the sensor structure aligned with the axis of the sample. The S_21_ curve of the sensor is measured using Agilent’s N5242A, and its overall detection platform is shown in Figure 21b.

### 5.2. Experimental Results

**Figure 22:**
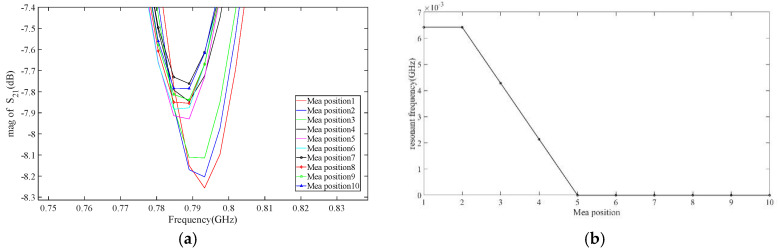
Frequency values of aircraft composite skin sample scanned in a straight line at each point. (**a**) Resonant frequency; (**b**) Frequency offset.

The nested multi-layer CSRR metasurface sensor scan along a straight line from point A to point B of the tested aircraft composite skin sample at a step size of 5 mm, scanning a total of 10 positions. The resonance frequency of each scanning point are measured by a vector network analyzer as shown in Figure 22a, and the resonant frequency values of each point are shown in Table 4. According to the analysis of Figure 22a and Table 4, the resonant frequencies of 10 points are located between 0.767605 GHz and 0.801854 GHz. The first and second points have higher frequencies, while the third and fourth points have excessive frequencies. The resonant frequencies of the fifth and subsequent points remain unchanged. Calculate the resonance frequency offset at 10 points as shown in Figure 22b. According to Figure 22b, it can be roughly determined that the defect is located at points 1 and 2, with a size of approximately 10 mm.

**Figure 23:**
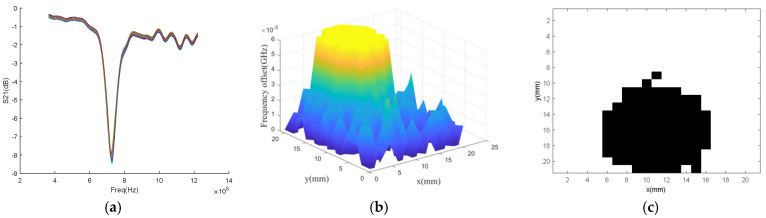
Frequency values of aircraft composite skin sample plane scanning at various points. (**a**) S_21_ parameter frequency response; (**b**) Three dimensional graph of frequency offset distribution; (**c**) Black and white image of frequency offset distribution.

Furthermore, defect in the tested aircraft composite skin sample is detected using nested multi-layer CSRR metasurface sensor for two-dimensional scanning with a scanning step of 1 mm. The S_21_ frequency response curves of each scanning point is shown in Figure 23a. Calculate the resonant frequency offset and obtain the three-dimensional frequency offset map of the tested aircraft composite skin sample as shown in Figure 23b. Denoise the frequency offset map, and the black and white frequency offset map is shown in Figure 23c. According to the analysis of Figure 23c, the defect diameter is about 10 mm, which is basically consistent with the actual defect, thus verifying the feasibility of nested multi-layer CSRR metasurface sensor detection.

## 6. Conclusions

In the paper, the new nested multi-layer CSRR structured metasurface sensor is proposed. Compared to traditional CSRR structures. On the basis of maintaining the overall size unchanged, add a resonant ring inward to reduce the resonant frequency. Compared with traditional CSRR structures, its sensing area has more concentrated and stronger energy. The designed multi-layer CSRR metasurface sensor is used for detecting internal defects in aircraft composite skin. Through HFSS simulation analysis, nested multi-layer CSRR metasurface sensors can detect defects with a diameter of 2 mm or more and a depth of up to 3 mm. Finally, the aircraft composite skin samples were tested and artificially manufactured defects were successfully detected. The defect location and size matched the actual defects, thereby verifying the effectiveness of the nested multi-layer CSRR metasurface sensor for aircraft composite skin defect detection.

## Figures and Tables

**Table 1:** Nested multi-layer CSRR structure size parameters.

Parameter	Unit (mm)
shape	square
$L_{1}$	12
$L_{2}$	12
***N***	10
$L_{3}$	4.4
$L_{4}$	4.4
w	0.2
s	0.2
g	0.2

**Table 2:** Material property parameters.

Material	Conductivity (S/m)	Relative Permittivity	Relative Permeability
longitude carbon fiber	$\left[\begin{array}{ccc} 20,000 & 0 & 0\\ 0 & 200 & 0\\ 0 & 0 & 200 \end{array}\right]$	$\left[\begin{array}{ccc} 3.26 & 0 & 0\\ 0 & 3.02 & 0\\ 0 & 0 & 3.26 \end{array}\right]$	1
transverse carbon fiber	$\left[\begin{array}{ccc} 200 & 0 & 0\\ 0 & 20,000 & 0\\ 0 & 0 & 200 \end{array}\right]$	$\left[\begin{array}{ccc} 3.02 & 0 & 0\\ 0 & 3.26 & 0\\ 0 & 0 & 3.26 \end{array}\right]$	1
resin matrix	0	4.4	1
air	0	1	1

**Table 3:** Comparison with other detection methods.

References	Type	Defect Detection Resolution
[17]	Ultrasonic	3 mm
[18,19]	eddy current	6 mm
The work	Metamaterial sensor	2 mm

**Table 4:** Resonant Frequency Values at Linear Scanning Points.

Scan Points	Resonant Frequency (GHz)
1 (A)	0.793292
2	0.793292
3	0.791152
4	0.789011
5	0.786871
6	0.786871
7	0.786871
8	0.786871
9	0.786871
10 (B)	0.786871

## Data Availability

The original contributions presented in this study are included in the article. Further inquiries can be directed to the corresponding author.
